# Growth-density inversion in *Escherichia coli* reveals superlinear, not sublinear, density dependence

**DOI:** 10.1371/journal.pbio.3003898

**Published:** 2026-07-16

**Authors:** James A. Orr, Kaleigh E. Davis, Alicia H. Williams, Jan Engelstädter, Daniel B. Stouffer, Andrew D. Letten

**Affiliations:** 1 School of the Environment, The University of Queensland, Brisbane, Australia; 2 Department of Integrative Biology, University of Guelph, Guelph, Canada; 3 Department of Evolutionary and Integrative Ecology, Leibniz Institute of Freshwater Ecology and Inland Fisheries (IGB), Berlin, Germany; University College London - Bloomsbury Campus: University College London, UNITED KINGDOM OF GREAT BRITAIN AND NORTHERN IRELAND

## Abstract

Density dependence is a core principle in ecology and evolution, and yet the shape of the relationship between per capita growth and population size remains contentious. Sublinear (decelerating) density dependence has been widely reported in empirical studies, but the apparent ubiquity of sublinearity is at odds with resource competition theory. Moreover, most estimates of density dependence suffer from underappreciated statistical biases and weak inference. Using a microbial, continuous-culture approach that bypasses the limitations of conventional methods by inverting the direction of causality, we report robust evidence for superlinear (accelerating) density dependence, in agreement with theory. These results underscore the need for concerted efforts to reconcile a widening disconnect between mechanistic and phenomenological approaches to inference on the shape of density dependence. Resolving this debate has wide implications for past and future research into species coexistence and ecosystem stability, and in conservation and natural resource modelling.

## Abstract 2

Sublinear density dependence has been widely reported empirically, but this is at odds with resource competition theory. This study uses a tightly controlled test in a microbial model system to reveal superlinear density dependence, aligning empirical evidence with theory.

## Introduction

Density-dependent population growth is a fundamental principle in ecology and evolution, shaping both the generation and maintenance of biodiversity. For over a century, scientists have used phenomenological models of density dependence to help understand, predict, and manage populations of animals, plants, and microbes [1–3]. The classic example is the logistic model, where per capita growth rate declines linearly with density. Few organisms, however, exhibit strictly linear density dependence. One pattern that is generally believed to predominate in nature is so-called ‘sublinear’ (i.e., decelerating) density dependence [3,4], where per capita growth rate declines most rapidly at low densities. It is less widely appreciated that pure sublinearity runs counter to the predictions of mechanistic models of competition, which typically generate superlinear (i.e., accelerating) or more complex patterns of density dependence [5–8]. The extent to which this tension reflects a flaw in the theory or the data remains unclear.

A central empirical challenge in estimating the shape of density dependence is that in most systems, it is impractical to hold densities constant while simultaneously observing resulting growth rates [5]. Exclusively studying organisms with discrete growth dynamics offers a partial solution, but the potential for decoupled effects of density on growth rate (e.g., due to delayed resource depletion) remains a problem [5,9]. Both experiments and observational time-series are also plagued by statistical issues, with sublinearity readily emerging as a simple artefact of regressing a noisy variable upon itself [10]. Sparse sampling can introduce a comparable bias [10], while inference from natural systems may also be compromised by the confounding effect of interspecific interactions [3]. Taken together, these empirical limitations cast doubt on the reliability of conventional methods for investigating density dependence.

To circumvent these problems, Abrams [5] proposed an alternative experimental approach that reverses the logic of traditional tests of density-dependence; instead of measuring per-capita growth rate over experimentally manipulated densities, the hypothetical experimenter manipulates harvest rates in order to observe their impact on equilibrium density [5]. In a continuously harvested population, per capita growth rate is equal to the harvest rate at equilibrium, and therefore equilibrium density can be treated as a function of growth rather than the other way around [5,9]. Inverting this function returns the familiar curve describing growth rate as a function of density, hence we term this technique ‘growth-density inversion’ (see Box 1 and the Supporting information for more details). While Abrams recognised the impracticalities of implementing this approach in most natural systems, he employed the same logic to deduce the emergent shape of density dependence arising from classical consumer-resource models [5,11]. Given saturating growth functions, consumer-resource models point to a greater prevalence of superlinear density dependence than the empirical data would suggest [5,6,8].

**Fig 1:**
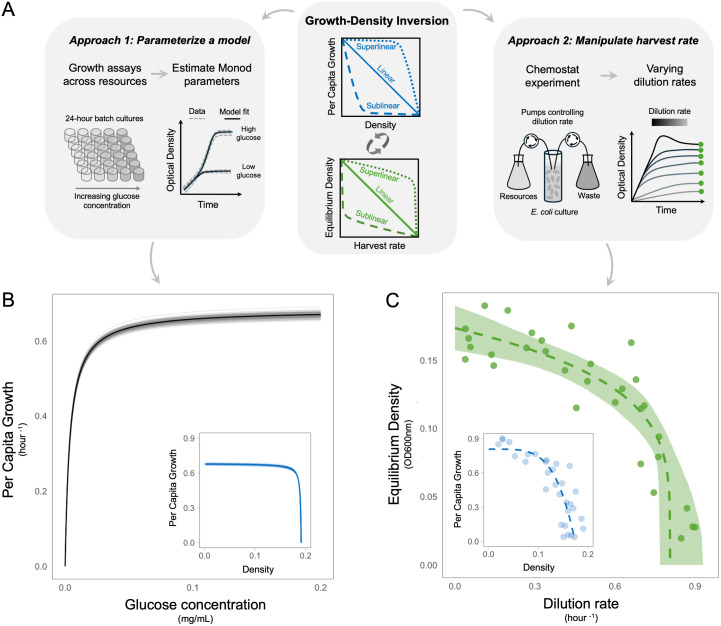
**(A)** Schematic of the empirical application of growth-density inversion, where the classic density dependence perspective (blue) is inverted (green) to overcome the challenges of manipulating densities. We first parametrised a consumer-resource model using batch culture growth assays (*Approach 1*) and performed growth-density inversion analytically (Box 1). Then, in a model-free empirical implementation of growth-density inversion, we experimentally manipulated harvest rate and quantified densities at equilibrium by varying the dilution rate in a chemostat system (*Approach 2*). **(B)** Parametrised Monod growth function with 100 posterior draws representing uncertainty. The inset shows the analytical prediction for the density dependence of this parametrised consumer–resource model. **(C)** Inverse θ-logistic model fitted to the experimental data with 95% credible intervals. Inset shows the same data and model with axes inverted to give the classic density dependence perspective. The data and code needed to generate this figure can be found at https://doi.org/10.5281/zenodo.20848361.

Building upon Abrams’ insights, we investigated the shape of density dependence in a minimal microbial model (*Escherichia coli*). This critically reductionist approach allows for direct observations of density dependence that are unobscured by interspecific interactions, environmental variability, immigration, statistical biases, and other confounding factors. We first used growth-density inversion to generate analytical predictions for the shape of density dependence via an empirically parametrised consumer-resource model (Box 1, Fig 1A *Approach 1*). We then conducted a stricter, model-free empirical implementation, using a classical experimental design (see [12]) to measure equilibrium densities under different dilution (i.e., harvesting) rates in continuous culture (Fig 1A *Approach 2*). Together, these two independent but complementary approaches combine the mechanistic insight afforded by a model-based prediction, with a fully empirical test of density dependence that bypasses the statistical and experimental artifacts of traditional methods.

**Box 1. Growth-density inversion of a consumer-resource model.**

Following Abrams [5], here we walk through the steps for analytical (cf. empirical) growth-density inversion. We start with a consumer-resource model describing the dynamics of one consumer with Monod (type II) growth on a single resource under continuous supply:

$$\begin{array}{c} \frac{dN}{dt}=N\left(\frac{\mu_{max}R}{k_{S}+R}-m\right)\;\frac{dR}{dt}=d(S-R)-\frac{\mu_{max}R}{k_{S}+R}QN \end{array}$$ (1)

where *N* is the consumer density, *R* is the resource density, $\mu_{max}$ is the maximum growth rate, $k_{S}$ is the half saturation constant, *m* is the mortality rate, *d* is the dilution rate, *S* is resource density in the supply, and *Q* is the quota (i.e., resources per consumer).

First we set the right hand sides of the equations to zero and solve for the equilibrium values of consumer ($N^{*}$) and resource ($R^{*}$). When the consumer is at equilibrium, $\frac{\mu_{max}R^{*}}{k_{S}+R^{*}}=m$, which we can rearrange in terms of the resource equilibrium density:

$$\begin{array}{c} R^{*}=\frac{m\;k_{S}}{\mu_{max}-m} \end{array}$$ (2)

Next we can rearrange the resource equilibrium equation (where $\frac{dR}{dt}=0$) in terms of the consumer equilibrium density:

$$\begin{array}{c} N^{*}=\frac{d\left(S-R^{*}\right)}{Q}\left[\frac{k_{S}+R^{*}}{\mu_{\max}R^{*}}\right] \end{array}$$ (3)

Finally, we substitute in the expression for $R^{*}$ (eq. 2) and simplify to complete the process of growth-density inversion by expressing consumer equilibrium density in terms of mortality rate:

$$\begin{array}{c} N^{*}=\frac{d}{\mu_{max}Q}\left[\frac{\mu_{max}S}{m}-\frac{m\;k_{S}}{\mu_{max}-m}-k_{S}\right] \end{array}$$ (4)

Examining the second partial derivative of $N^{*}$ with respect to *m* reveals the qualitative shape of density dependence for the consumer.

$$\begin{array}{c} \frac{\partial^{2}N^{*}}{\partial m^{2}}=\frac{2d}{Q}\left[\frac{S}{m^{3}}-\frac{k}{(\mu_{\max}-m{)}^{3}}\right] \end{array}$$ (5)

**Fig 2:**
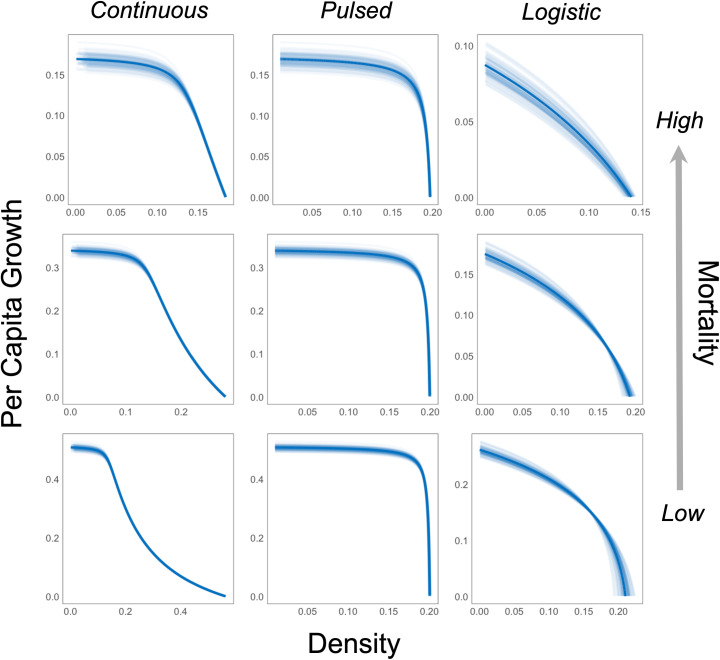
Predicted density dependence across three resource supply regimes (columns) and three baseline mortality rates (rows). Thick lines show predictions using median parameter estimates and thin lines are 100 posterior draws representing uncertainty. The data and code needed to generate this figure can be found at https://doi.org/10.5281/zenodo.20848361.

When $\frac{S}{m^{3}}>\frac{k}{(\mu_{\max}-m{)}^{3}}$, the second derivative is positive, and the function is concave up (sublinear). When $\frac{S}{m^{3}}<\frac{k}{(\mu_{\max}-m{)}^{3}}$ the second derivative is negative and the function is concave down (superlinear). Given $\mu_{\max}>m$, it follows that density dependence will be superlinear at low density (high *m*) and sublinear at high density (low *m*) (first column of Fig 2). This inflection from super- to sublinearity reflects the changing balance in the weighting of the type II functional response (causing superlinearity) and the continuous resource dynamic (causing sublinearity) in determining the shape of density dependence. Abrams and others have referred to this influence of the shape of the resource’s own density dependence on the shape of the consumer’s density dependence as the “principle of the inheritance of the curvature” [5,6]. Indeed, a type I (linear) functional response yields purely sublinear consumer density dependence under continuous resource supply, but linear consumer density dependence given logistic or pulsed resource supply.

In this study, we manipulated chemostat dilution rate to experimentally control population growth rate (Fig 1C). When *m* = *d*, the second partial derivative becomes,

$$\begin{array}{c} \frac{\partial^{2}N^{*}}{\partial m^{2}}=\frac{2k\mu_{\max}}{Q(m-\mu_{\max}{)}^{3}}, \end{array}$$ (6)

the consequence of which is that the contribution of the chemostat resource supply to density dependence is cancelled out, and density dependence is purely superlinear. Nevertheless, the influence of alternative resource supply dynamics can still be inferred from an empirically parametrised model (as in Fig 2).

## Results and discussion

We used 143 independent growth assays for *E. coli* strain MG1655 across a range of initial glucose concentrations to parametrise a consumer-resource model assuming Monod growth dynamics (Fig 1B). The choice to use a Monod (type II) functional response, rather than an alternative response (e.g., type I or III), is supported by extensive empirical evidence showing *E. coli*’s growth saturates rapidly on limiting glucose [13–15]. Solving the model for equilibrium density assuming a range of continuous dilution (i.e., harvesting) rates predicted an acutely superlinear density-dependent function for *E. coli* (Box 1, inset in Fig 1B). To test this prediction, we then ran a series of independent chemostat experiments, growing replicate lines under equivalent dilution rates to those investigated analytically (until they established a stable equilibrium). Next, following convention in the density dependence literature, we used a θ-logistic model to provide inference on the shape of the density dependence observed across the chemostat experiments. More precisely, to align with growth-density inversion, we fitted an inverse θ-logistic model to equilibrium density as a function of dilution rate, where θ<1, θ>1 and θ=1 correspond to sublinear, superlinear, and linear density dependence, respectively (see Methods and Supporting information for further methodological details). The experimental results aligned with the predictions of the consumer-resource model. More specifically, we found compelling evidence for superlinear density dependence, with θ=4.55 (95% credible intervals: 2.32–8.38) (Fig 1C).

The role of the consumer’s functional response in determining the shape of emergent density dependence is conspicuous in Fig 1B. Abrams’ principle of “inheritance of the curvature" posits that a consumer’s density dependence will also be influenced by the shape of density dependence of the resource [5,11]. In contrast to systems where resources grow logistically or enter in periodic pulses, continuous resource supply represents one of the few theoretical conditions under which regions of sublinear density dependence may arise [5,6,8]. In our experimental system, however, adjusting chemostat dilution rate, and therefore harvest rate, also meant changing the resource supply rate, a consequence of which is that the independent effect of the resource supply dynamic on the emergent shape of density dependence is cancelled out. We therefore also investigated the predictions of the empirically parametrised model under three alternative resource supply regimes (continuous, pulsed, and logistic) and three baseline mortality rates (25%, 50%, and 75% of the maximum growth rate). Under pulsed and logistic supply, the model predicts exclusively superlinear density dependence (centre and right columns in Fig 2). Under continuous resource supply, the model predicts a switch from superlinearity to sublinearity as density increases (left column in Fig 2), with the latter region only being conspicuous at low mortality rates.

The predicted absence of sublinearity at low densities (under any combination of resource dynamic and mortality) stands in contrast with a large body of theoretical and empirical research whose conclusions derive from models characterised by sublinear density dependence [16,17]. In a recent study [3], sublinearity at low density was a crucial factor fostering a presumptive positive relationship between diversity and stability [18]. Our findings are also at odds with the assumptions of sublinearity that underpin many population models that inform conservation and resource management decisions [19,20]. It may be tempting to attribute this disconnect to the ecological simplicity of our bacterial study system or as a phenomenon that is more broadly unique to microbes. Indeed, a recent analysis of diverse microbial time-series (employing a novel statistical approach) also found no evidence for sublinear growth [21]. Nevertheless, we are unaware of any arguments from first principles why increasing organismal or system complexity itself, or mechanisms only present in multicellular organisms, should drive a universal switch from superlinearity to sublinearity. On the contrary, given the prevalence of saturating functional responses across diverse organisms [22], a reasonable prediction deriving from the principle of inheritance of the curvature [5] is that density dependence will only become increasingly superlinear with each jump in trophic level.

To our knowledge, this is the first study to investigate density dependence using empirically parametrised consumer-resource models in combination with experimental growth-density inversion. Nevertheless, our experimental design echoes early microbiological chemostat studies that tested the predictive power of the Monod model under continuous resource supply, including several that directly measured equilibrium density as a function of dilution rate. For example, Herbert and colleagues [12] compared observations on *Klebsiella aerogenes* with a model partially parametrised from independent batch culture, while Contois [23] even flipped the axes (as in Fig 1C inset) to argue for independent contributions of resource supply and density to specific growth rate in *Enterobacter cloacae*. Both sets of authors observed strongly superlinear relationships between density and dilution rate, as did later studies in *E. coli* [24] and in yeast (*Saccharomyces cerevisiae*) [25]. Comparable experiments in algal systems provide evidence for more linear (but not sublinear) relationships between dilution and cell density [26,27]. While none of these studies considered the consequences of alternative resource supply regimes (as in Fig 2), taken together they underscore the need for greater scrutiny of inference of sublinearity deriving from time-series data.

We deliberately used a single carbon source (glucose) at low concentrations in the chemostat experiments in order to minimise the likelihood of multiple limiting factors affecting observed density dependence. In a recent study referencing our results, the authors argue that superlinear density dependence only emerges as resources become limiting at densities close to equilibrium [28]. Away from equilibrium at intermediate densities, the authors report sublinear growth in *E. coli*, and suggest that factors unrelated to resource limitation (e.g., quorum sensing or toxins) play a dominant role. Notwithstanding the acknowledged obstacles to accurately inferring density dependence from time-series data [10], we posit that their observations may in fact be entirely compatible with multiple resource limitation [11], with sublinearity emerging as a feature of the transition from one superlinear region to another as different resources become limiting. This is to say it may be reasonable to construe superlinearity as the basic building block or fundamental unit of density dependence. Nevertheless, allowing for increasing abiotic and biotic complexity (e.g., behavioural switching or multiple life stages) can yield richer patterns of density dependence, comprising both super‑ and sublinear regions [11].

The qualitative alignment we observe between model-predicted and experimentally observed density dependence (Fig 1B and Fig 1C insets, respectively) makes a compelling case for the explanatory power of even simple consumer-resource models, particularly given that the model was parametrised using independent batch culture data. That being said, the higher maximum growth rate observed under continuous culture conditions (y-intercept in Fig 1C inset) suggests a potential plastic shift to a faster-growing phenotype at high dilution rates. This deviation between model prediction and empirical result was also observed by Herbert and colleagues [12], who hypothesised that it might be partially driven by imperfect mixing in chemostats. More broadly, we recognise that besides phenotypic plasticity and spatial heterogeneity, there are many biological processes (e.g., dispersal, species interactions) that could drive deviations from the basic predictions of Monod dynamics. However, we are aware of no *a priori* reasons why those deviations should universally drive density dependence towards sublinearity.

## Conclusions

In this study, we found strong evidence for superlinear density dependence that aligns with predictions from mechanistic theory; predictions that are general and taxonomically agnostic. When considered alongside the biases introduced by conventional methods for estimating density dependence, we contend that the evidence supporting sublinear density dependence as a universal phenomenon may be less robust than commonly assumed. Nevertheless, we cannot yet rule out the possibility that sublinearity emerges more readily in other taxa, or that a general mechanistic basis for sublinearity is not waiting to be discovered. To the extent they are experimentally feasible, further applications of growth-density inversion to reliably identify the shape of density dependence in more complex taxa and systems would be immensely valuable. Resolving the debate around the shape of density dependence carries potentially significant implications, not only for our fundamental understanding of ecosystem stability and the maintenance of biodiversity, but also for the development of reliable population models that inform conservation and resource management strategies.

## Methods

We used 143 independent batch-culture growth assays of the model bacteria *E. coli* (MG1655) to parametrise a consumer-resource model where, following the prevailing literature on bacterial growth dynamics, resource-dependent growth followed a Monod relationship (i.e., growth rate saturates at high resource concentrations) [15]. Populations were grown in M9 media at 10 different glucose concentrations ranging from 0%–0.01% glucose in a 96-well plate maintained at 37 °C. Optical density measurements were made every minute in an Epoch 2 plate reader over 24 hours. We estimated the parameters of the consumer-resource model via Bayesian inference using the sample() method from the cmdstanr package v0.9.0 [29]. We sampled over four chains with 1,000 warmup and 1,000 post-warmup Hamiltonian Monte Carlo iterations, resulting in a total of 4,000 posterior samples. We solved the initial-value problem using the ode_rk45() method in Stan [30,31]. The full Bayesian description of our model, including prior distributions is available in the Supporting information. We then used those parameters to predict the shape of density dependence for *E. coli* in continuous culture following Abrams’ analytical approach (Box 1, [5,11]).

We empirically tested this prediction by growing *E. coli* populations in 30mL chemostats supplied with 0.05% glucose M9 media. A relatively low concentration of glucose was chosen for the supply media to ensure that only glucose, and not some other component of the M9 media, was limiting population growth. Although the concentration of glucose inside the reactors themselves varied over the experiment depending on dilution rate and bacterial population dynamics, it would typically be orders of magnitude lower than the concentration of glucose in the supply and would therefore be well within the range of glucose concentrations tested in the batch culture assays. Five experimental replicates were performed using a “Chi.Bio” chemostat system comprised of eight 30 mL reactors connected to a series of peristaltic pumps. The reactors were held at constant temperature with continuous stirring and measurement of optical density (600 nm) at 1-min intervals. In each run of the experiment, we tested six different dilution rates (that varied within and across experimental replicates), in addition to one methodological control (*E. coli* grown at the same dilution rate across experimental replicates) and one biological control (sterile media). The inflow rates of the reactors were manipulated to experimentally control dilution rate (and therefore bacteria harvest rate). There was a small level of unavoidable variability in the dilution rates of reactors that were intended to have identical dilution rates (as for the methodological controls) due to slight differences in the efficiency of each reactor’s pumps. However, methodological controls (all with very similar low dilution rates) showed similar growth dynamics and equilibrium densities over the four-day experiments (Fig A(a) in S1 Appendix). To complement the frequent, but less precise, optical density readings of the “Chi.Bio” reactors, the chemostats were sampled daily and more precise optical density measurements were taken using an Epoch 2 plate reader. The frequent “Chi.Bio” optical density readings (i.e., Fig A(b) in S1 Appendix) were used to infer when populations were at equilibrium, while the more precise plate reader optical density measurements (i.e., Fig A(a) in S1 Appendix) were used for all analyses.

The inverse θ-logistic model was used as an inference tool to quantify the shape of the density dependence observed in the chemostat experiments using (i) a Bayesian model implemented in Stan via the brms R package [32], which used MCMC and constrained the estimated maximum growth rate to be greater than the highest observed growth rate, and (ii) a grid-based Bayesian approach, which prevented predictions of negative densities so was well-defined over the full parameter space (Fig 1B). The model implement in Stan was fit with a Gamma distribution and an identity link function, since equilibrium densities cannot be negative. Broad but biologically plausible priors were chosen for *r* (uniform distribution between 0.5 and 1.5) and *K* (uniform distribution between 0.1 and 0.5) based on growth rates and densities previously observed in this system (i.e., the batch culture growth assays and pilot chemostat studies). We purposely chose weak priors for *r* and *K* to highlight that inferences about the shape of density dependence were being driven by the data rather than by the priors. We set uninformative priors for θ (uniform distribution between 0.1 and 10), ranging from highly sublinear to highly superlinear. (Note that as θ is an exponent, values of 0.1 and 10 are approximately equally distant from the logistic case of θ=1 on the multiplicative scale.) For the grid-based approach, which was feasible given the low dimensionality of the inverse θ-logistic model, posterior probabilities for all combinations of 50 values of *K*, 50 values of θ, 50 values of *r*, and 20 values of the standard deviation of the residual errors was calculated. Randomly drawing combinations of parameters from the grid weighted by their posterior probability allowed us to obtain posterior draws and posterior predictive distributions.

Finally, the parametrised consumer-resource model was used to predict the shape of density dependence under continuous, pulsed, and logistic resource supply regimes. Median parameter estimates were used to make predictions and posterior draws were used to represent uncertainty. We plotted the observed density dependence for each of the three resource supply regimes under baseline mortality rates of 25%, 50%, and 75% of the maximum growth rate to illustrate the importance of baseline mortality rates in determining the shape of density dependence. Further details of all experiments and analyses are available in the Supporting Information and in the accompanying R notebooks at https://doi.org/10.5281/zenodo.20848361.

## Supporting information

S1 Appendix Fig A in S1 Appendix. Optical density data from the chemostat experiment. **(a)** The chemostats were sampled once per day during the experiment and the optical density of these samples was precisely quantified using an Epoch 2 plate reader. The point symbols represent the five experimental replicates, the colour of the points represents the day (one to four) the sample was taken, and grey vertical bars connect samples taken from the same reactor. Methodological controls from different experimental replicates (apart from dd6 whose pump failed and was therefore excluded) showed the same overshooting growth dynamics and had very similar equilibrium densities. **(b)** The chemostats contain optical density readers that are less precise than a plate reader, but that quantify the optical density of the culture inside the reactor every minute. Timeseries of the optical densities on a logarithmic scale over the four day experiment are shown for three reactors that represent high, medium, and low dilution rates. Negative optical density values (arising from measurement noise near the detection limit) are not shown due to the logarithmic scaling but are available in the raw data. Points show optical density readings per minute and the colours correspond to dilution rate. The data and code needed to generate this figure can be found at https://doi.org/10.5281/zenodo.20848361. **Section A in S1 Appendix.** Modelling resource-dependent consumer growth. **Section B in S1 Appendix.** Growth-density inversion. **Section C in S1 Appendix.** Experimental procedure. **Section D in S1 Appendix.** Estimating the shape of observed density dependence. **Section E in S1 Appendix.** Density-dependence predictions for other resource dynamics.
(PDF)
