# Supplementary material for: Growth-density inversion in Escherichia coli reveals superlinear, not sublinear, density dependence
Source: PLoS Biol. 2026 Jul 16;24(7):e3003898. doi: 10.1371/journal.pbio.3003898 (PMC13395313; doi:10.1371/journal.pbio.3003898)
Supplement: S1 Appendix — Optical density data from the chemostat experiment. (a) The chemostats were sampled once per day during the experiment and the optical density of these samples was precisely quantified using an Epoch 2 plate reader. The point symbols represent the five experimental replicates, the colour of the points represents the day (one to four) the sample was taken, and grey vertical bars connect samples taken from the same reactor. Methodological controls from different experimental replicates (apart from dd6 whose pump failed and was therefore excluded) showed the same overshooting growth dynamics and had very similar equilibrium densities. (b) The chemostats contain optical density readers that are less precise than a plate reader, but that quantify the optical density of the culture inside the reactor every minute. Timeseries of the optical densities on a logarithmic scale over the four day experiment are shown for three reactors that represent high, medium, and low dilution rates. Negative optical density values (arising from measurement noise near the detection limit) are not shown due to the logarithmic scaling but are available in the raw data. Points show optical density readings per minute and the colours correspond to dilution rate. The data and code needed to generate this figure can be found at https://doi.org/10.5281/zenodo.20848361. Section A in S1 Appendix. Modelling resource-dependent consumer growth. Section B in S1 Appendix. Growth-density inversion. Section C in S1 Appendix. Experimental procedure. Section D in S1 Appendix. Estimating the shape of observed density dependence. Section E in S1 Appendix. Density-dependence predictions for other resource dynamics. (PDF) [file pbio.3003898.s001.pdf]

Supporting Information for

Growth-density inversion in *E. coli* reveals superlinear, not sublinear, density dependence

James A. Orr[1], Kaleigh E. Davis[1,2], Alicia H. Williams[1], Jan Engelstädter[1], Daniel B. Stouffer[3], and Andrew D. Letten[1]

[1]School of the Environment, The University of Queensland, Brisbane, Queensland 4072, Australia
[2]Department of Integrative Biology, University of Guelph, Guelph, Ontario, Canada
[3]Department of Evolutionary and Integrative Ecology, Leibniz Institute of Freshwater Ecology and Inland Fisheries (IGB), Berlin, Germany

Contents

A Modelling resource-dependent consumer growth **2**
A.1 Batch culture data 2
A.2 Model formulation and parameterization 2

B Growth-density inversion **3**

C Experimental procedure **4**
C.1 Calculating dilution rates 5
C.2 Determining when populations were at equilibrium 5
C.3 Observed growth dynamics 7

D Estimating the shape of observed density dependence **7**

E Density-dependence predictions for other resource dynamics **9**

A Modelling resource-dependent consumer growth

A.1 Batch culture data

Growth curves were obtained for *E. coli* strain MG1655 across a range of initial glucose concentrations. For each replicate, an overnight culture was grown at 37 °C in M9 media supplemented with 0.05% glucose for 18 hours. 1mL of culture was then pelleted and resuspended in 200uL M9 0% glucose in order to remove any residual glucose. 2μL of this resuspension was subsequently inoculated into 178μL of M9 with ten different glucose concentrations (0, 0.0005, 0.001, 0.002, 0.003, 0.004, 0.005, 0.006, 0.008, 0.01 % glucose) in a 96-well plate maintained at 37 °C. Optical density measurements were made every minute in a Epoch 2 plate reader over 24 hours. Owing to the appearance of two distinct growth phases (likely attributable to a diauxic shift from the primary glucose substrate to a carbon intermediate), we truncated the growth curve to only include the first, glucose limiting phase of growth. Each glucose concentration was replicated 15 times for a total of 150 individual assays (seven were subsequently excluded due to contamination or irregular OD measurements).

A.2 Model formulation and parameterization

Based on the prevailing literature, we assumed that *E. coli* in batch culture should show resource-dependent growth that follows a Monod or Michaelis–Menten relationship (i.e., growth rate saturates at high resource concentrations) [1]:

$$\frac{dN}{dt} = \frac{\mu_{\max} R}{k_S + R} N \,, \tag{S1}$$

where N is the consumer biomass density, R is the resource concentration, $\mu_{\max}$ is the maximum growth rate and, and k_S is the half-saturation constant. In parallel to the equation for consumer growth, there is a corresponding equation for resource depletion given by

$$\frac{dR}{dt} = -\frac{1}{\upsilon} \frac{\mu_{\max} R}{k_S + R} N \,, \tag{S2}$$

where υ is the yield of consumer density produced per one unit of resource concentration taken up (note that yield is $1/Q$, where Q is the quota from the main text).

Upon noting that the quantity $K(t) = N(t) + \upsilon R(t)$ is actually constant for all times because $\frac{dK}{dt} = \frac{dN}{dt} + \upsilon \frac{dR}{dt} \equiv 0$ given both Eq. S1 & S2, it is possible to use a "conservation approach" to describe the consumer dynamics [2]. This allows us to eliminate R from Eq. S1 to give

$$\frac{dN}{dt} = \frac{\mu_{\max} (K - N)}{\upsilon\, k_S + K - N} N \,. \tag{S3}$$

This conversation approach further implies that $K \equiv N_0 + \nu R_0$, where N_0 is the initial consumer biomass density and R_0 is the initial resource concentration. The full set of parameters needed to predict growth dynamics is $\{N_0, R_0, \mu_{\max}, k_S, \upsilon\}$.

Since the initial inoculum densities deriving for each of the 15 overnight cultures potentially varied between themselves, we treated each of these 15 values as additional unknowns when fitting the model to the observed data [3]. Optical density measurements are also subject to variation due directly to the M9 growth media. To account for this as well

as non-independence across observations, we allow the OD due to growth media to vary randomly across 96-well plates and across wells nested within plates using a hierarchical model. We furthermore assumed that the total optical density in a well was the sum of the optical density due to the growth media and the optical density due to the consumer biomass.

We estimated the model parameters via Bayesian inference using the `sample()` method from the `cmdstanr` package v0.9.0 [4] in `R` v4.5.1 [5]. We sampled over four chains with 1000 warmup and 1000 post-warmup Hamiltonian Monte Carlo iterations, resulting in a total of 4000 posterior samples. To promote chain convergence, we set the `max_treedepth` parameter to 20 and the `adapt_delta` parameter to 0.999. We solved the initial-value problem using the `ode_rk45()` method in Stan [6]. All unknown parameters were constrained to be positive in order to keep the model biologically sensible. The full Bayesian description of our model including prior distributions is

$$\mathrm{OD}_{pwt} \sim \mathrm{Lognormal}\left(\ln \lambda_{pwt}, \sigma_{\mathrm{OD}}\right) \tag{S4}$$

$$\lambda_{pwt} = N_{pwt} + b_{pw} \tag{S5}$$

$$N_{pwt} = \texttt{ode\_rk45}\left(N_{0,p}, t, K_{pw}, \mu_{\max}, k_S, \upsilon\right) \tag{S6}$$

$$K_{pw} = N_{0,p} + \upsilon R_{0,pw} \tag{S7}$$

$$\ln b_{pw} = \beta_{pw} \tag{S8}$$

$$\{\ln N_{0,1}, \ldots, \ln N_{0,15}\} \sim \mathrm{Normal}(0,1) \tag{S9}$$

$$\{\ln \mu_{\max}, \ln k_S, \ln \upsilon\} \sim \mathrm{Uniform}(-\infty, \infty) \tag{S10}$$

$$\beta_0 \sim \mathrm{Uniform}(-\infty, \infty) \tag{S11}$$

$$\beta_p \sim \mathrm{Normal}(\beta_0, \sigma_p) \tag{S12}$$

$$\beta_{pw} \sim \mathrm{Normal}(\beta_p, \sigma_w) \tag{S13}$$

$$\{\sigma_{\mathrm{OD}}, \sigma_p, \sigma_w\} \sim \text{Student-}t(3, 0, 2.5) \tag{S14}$$

where for convenience we have introduced the subscript p to denote the 96-well plate, the subscript w to denote the well in that plate, and the subscript t to denote the time elapsed at the moment of estimating optical density.

B Growth-density inversion

Traditional tests of density dependence, where species densities are manipulated (often to unrealistic levels) and resulting growth rates are quantified, suffer from time delay issues [7, 8]. Specifically, delayed effects of density on growth rate distorts the shape of density dependence and keeping densities fixed is impossible for many systems. Given these challenges, an alternative test of density dependence was proposed by Peter Abrams [7, 9]. This intuitive approach, which we call "growth-density inversion", reverses the logic of traditional tests of density-dependence by manipulating per capita growth and quantifying resulting densities. In practice, this entails varying harvest rate and quantifying densities once equilibrium is reached (when harvest is equal to growth).

Here, we studied the density dependence of *Escherichia coli* by manipulating the dilution rate of chemostats and quantifying bacterial population density at equilibrium (when growth rate equals dilution rate). Bacterial populations growing in chemostats is an ideal first empirical application of the growth-density inversion approach as steady state

equilibria are reached (cycling population dynamics would complicate the analysis), and the short generation time of microbes facilitates observation of population density responses to changes in per capita growth rates.

In Abrams' analytical work, density dependence is studied by varying "neutral parameters", which only directly impact a focal species [9]. However, experimentally manipulating a "neutral parameter" of the bacteria in a chemostat system, such as harvest rate, is a technical challenge. While dilution rate is not a "neutral parameter", in the sense that it directly impacts both the bacteria and the resources, it offers a feasible way to manipulate bacteria harvest rate, and we can analytically control for the effect it has on resource dynamics. A consumer's density dependence is influenced by the shape of its functional response and by the density dependence of the resource it consumes [7, 10, 11, 12]. By manipulating dilution rate we effectively observed how the bacteria's functional response shapes their density dependence independently of resource dynamics.

C Experimental procedure

Five experimental replicates were performed using a "Chi.Bio" chemostat system comprised of eight 30mL reactors connected to a series of peristaltic pumps. The reactors were held at constant temperature with continuous stirring and measurement of optical density measurement (600nm) at 1-minute intervals. In each run of the experiment, we tested six different dilution rates (that varied within and across experimental replicates), in addition to one methodological control (*E. coli* grown at the same dilution rate across experimental replicates) and one biological control (sterile media). Due to slight differences in the efficiency of each reactor's pumps, there was some uncontrollable variability in dilution rates of reactors. This was why we followed a regression-style design for the dilution treatment in the experiment rather than trying to obtain exact replicates of a fixed set of dilution rates.

The day before an experimental replicate, the reactors, tubing, and media bottles of the chemostat system were autoclaved, 0.05% glucose M9 media was prepared, and an overnight culture of *E. coli* was set up at 37 °C shaking at 180 rpm. Once the overnight culture had grown for 18 hours, reactors with 20mL media were inoculated with 1mL of the overnight culture (or 100μL in the first two experimental replicates). Although the inoculum size was increased in the later three experimental replicates in an effort to decrease the time taken for populations to reach equilibrium, we did not observe sizeable differences in time to equilibrium. Furthermore, we note that differences in inoculum size between the first two and the latter three experimental replicates did not impact equilibrium densities (Fig 1). All reactors were set to the same temperature (37 °C), stirring rate, and outflow rate. The inflow rates of the reactors varied in order to experimentally manipulated dilution rate (and therefore bacteria harvest rate). When the inflow pumps ran, fresh media entered the reactors and raised the level of the liquid above the outflow port. After several minutes of stirring, the outflow pumps ran until the level of the liquid in all reactors returned to the level of the outflow port. The inflow and outflow pumps ran every 20 minutes at fixed dilution rates that varied depending on inflow rates.

The chemostats were sampled each day for up to four days and the experiments ran until the populations were inferred to have reached equilibrium (based on the live optical

density read-out). The higher the dilution rate, the longer it took the populations to reach equilibrium. For sampling, 0.5ml of liquid was collected from the outflow ports of the reactors using syringes. 200μL of this was used to obtain higher precision optical density readings with a plate reader and 100μL was diluted and then plated onto LB agar for colony counting.

C.1 Calculating dilution rates

In classic chemostat theory, the dilution rate is defined as the volume of fresh media supplied per unit time divided by the volume of the culture. This assumes continuous flow, with instantaneous inflow and outflow, such that the volume of the culture is constant. In practice, however, chemostat systems often have discrete pulses of inflow and outflow, resulting in transient variation in culture volume. In our system, fresh media was added every 20 minutes and there was approximately two minutes intentionally left between inflows and outflows to allow for thorough mixing.

To calculate a continuous dilution rate in our system with discrete pulses we calculated the exponential decay rate that would have produced the same net dilution over a full inflow-outflow cycle. A single inflow pulse dilutes the population as follow:

$$N_1 = N_0 \cdot \frac{V_0}{V_0 + V_{in}} \tag{S15}$$

where V_0 is the baseline culture volume (21mL) and V_{in} is the inflow volume (equal to the outflow volume, which we measured daily). We can then match this discrete dilution factor to a continuous exponential decay model over a full cycle:

$$N_0 \cdot \frac{V_0}{V_0 + V_{in}} = N_0 \cdot e^{-d\tau} \tag{S16}$$

where τ is the length of the inflow-outflow cycle (20 minutes) and d is the dilution rate. After rearranging we have:

$$d = \frac{\log(\frac{V_0+V_{in}}{V_0})}{\tau} \tag{S17}$$

This allowed us to obtain a dilution rate that we could use for continuous-time modelling that reflected the effects of the discrete inflow and outflow pulses in our system.

C.2 Determining when populations were at equilibrium

Although samples were taken every day, only samples taken when populations were at equilibrium were used to estimate the shape of density dependence. Three biological phenomena needed to be considered when determining if a population had reached equilibrium: 1) populations under higher dilution rates took longer to reach equilibrium; 2) populations under the lowest dilution rates exhibited overshooting dynamics where density initially exceeded their equilibrium density before settling back down; 3) there was a risk of adaptive evolution to the different dilution rates through the course of the experiment. The growth dynamics described in the first two points above can be seen by

comparing daily samples from the same reactors (Fig 1A) or by comparing optical density time series from reactors under different dilution rates (Fig 1B).

Based on these factors, we used simple heuristics to determine when populations were at equilibrium. For the lowest dilution rates, samples taken on day four were considered at equilibrium to account for the overshooting dynamics (this was in line with previous work with this system). For the intermediate dilution rates, samples taken on days one and two were considered at equilibrium as there was no overshooting dynamics but there was a risk of evolution to experimental conditions (as evidenced by jumps in optical density after a period of stabilisation). For the highest dilution rates, samples taken on days three and four were considered at equilibrium as populations were still increasing on days one and two. No samples taken after day four were used due to the increased risk of evolutionary change. All dilution rates below 0.1 mL/hour/mL were treated as "low" dilution rates; optical density always decreased from day one to day four in these populations (overshoot). All dilution rates above 0.65 mL/hour/mL were treated as "high" dilution rates; optical density always increased from day one to days three/four in these populations. Dilution rates between 0.1 and 0.65 mL/hour/mL were treated as "intermediate" dilution rates; populations reached equilibrium by day one according to the "Chi.Bio" timeseries.

Five experimental replicates each with eight reactors gave us forty individual reactors. After removing the biological controls (no bacteria present), a reactor whose pump failed, and reactors that were not sampled on the days when their populations were considered at equilibrium, we were left with 31 reactors that had at least one sample taken when populations were at equilibrium. 15 reactors had two samples taken (on two separate days) when populations were at equilibrium, giving 46 observations in total. For these 15 reactors, we took the average optical densities of the two samples taken. As demonstrated in the R notebooks at `https://doi.org/10.5281/zenodo.20848361`, our results are insensitive to these data processing choices. Indeed, broadly ignoring the heuristics (e.g., by only considering samples from days three or four), changing the cutoff for what was considered "low", "intermediate", or "high" dilution rates, or using the first or last equilibrium sample rather than the average, all return data showing superlinear density dependence.

C.3 Observed growth dynamics

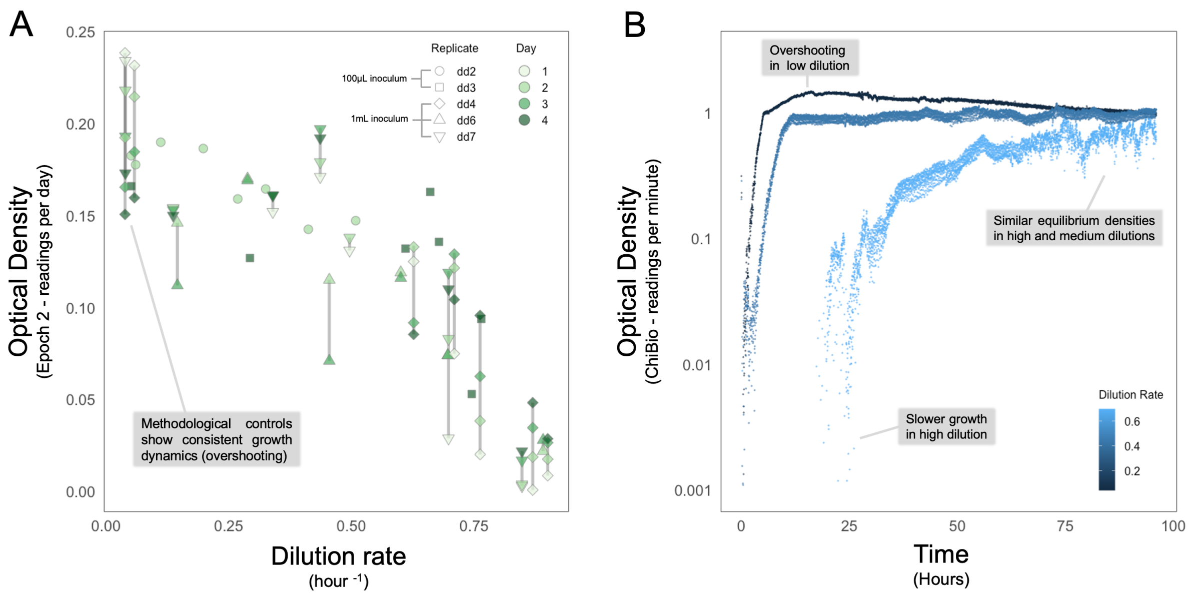

Fig A: **(a)** The chemostats were sampled once per day during the experiment and the optical density of these samples was precisely quantified using an Epoch 2 plate reader. The point symbols represent the five experimental replicates, the colour of the points represents the day (one to four) the sample was taken, and grey vertical bars connect samples taken from the same reactor. Methodological controls from different experimental replicates (apart from dd6 whose pump failed and was therefore excluded) showed the same overshooting growth dynamics and had very similar equilibrium densities. **(b)** The chemostats contain optical density readers that are less precise than a plate reader, but that quantify the optical density of the culture inside the reactor every minute. Timeseries of the optical densities on a logarithmic scale over the four day experiment are shown for three reactors that represent high, medium, and low dilution rates. Negative optical density values (arising from measurement noise near the detection limit) are not shown due to the logarithmic scaling but are available in the raw data. Points show optical density readings per minute and the colours correspond to dilution rate. The data and code needed to generate this figure can be found at `https://doi.org/10.5281/zenodo.20848361`.

D Estimating the shape of observed density dependence

The θ-logistic model is the canonical model for describing the shape of density dependence in ecology [13, 9, 14]. It extends the logistic model by including an additional parameter to control the non-linearity of the relationship between per capita growth and density:

$$g = r\left(1 - \left(\frac{N}{K}\right)^{\theta}\right) \tag{S18}$$

where $g = \frac{1}{N}\frac{dN}{dt}$ is the per capita growth, N is the population density, r is the maximum (intrinsic) growth rate, K is the carrying capacity, and θ controls the shape of density dependence. When $\theta > 1$, density dependence is superlinear; when $\theta < 1$, it is sublinear; and $\theta = 1$ recovers the classic logistic model. For our "Growth-Density Inversion"

experimental approach, per capita growth (dilution rate) was our explanatory variable, and density was our response variable. We therefore fit an inverse θ-logistic model to our data [7], which explained equilibrium density as a function of per capita growth:

$$N^* = K\left(1 - \frac{g}{r}\right)^{\frac{1}{\theta}} \tag{S19}$$

We first used the brms R package [15] to fit the inverse θ-logistic model (Eq. S19) to our data with a Gamma distribution and an identity link function since equilibrium densities cannot be negative. Broad but biologically plausible priors were chosen for r (uniform distribution between 0.5 and 1.5) and K (uniform distribution between 0.1 and 0.5) based on growth rates and densities previously observed in this system. We set very loose priors for θ (uniform distribution between 0.1 and 10) ranging from highly sublinear to highly superlinear as this was the key parameter we were interested in estimating from the data.

The posterior estimates of this model were: $K = 0.20$ (95% credible interval: 0.17 to 0.24), $r = 0.93$ (0.90 to 0.99), and $\theta = 1.95$ (1.31 to 2.71), indicating superlinear density dependence. Although the posterior predictive checks were reasonably good, the posterior distribution of r was bounded and negatively correlated with θ. As seen in Eq. S19, the model becomes undefined when the maximum growth rate (r) is less than the largest observed dilution rate (g). Thus, the model cannot explore the region of parameter space where $r < g$. Incorporating measurement error in g, log-transforming the model, or reparametrizing the model could not resolve this boundary issue.

To overcome this limitation in the fitting of the model, we reverted to a grid-based Bayesian approach that was feasible given the low dimensionality of our model. We slightly modified Eq. S19 by bounding the function at $N^* = 0$ when $g \geq r$:

$$N^* = K\left(\max\left\{0, 1 - \frac{g}{r}\right\}\right)^{\frac{1}{\theta}} \tag{S20}$$

This equation gives the globally stable equilibrium population size, which is given by Eq. S19 for $r > g$ and zero otherwise. Thus, Eq. S20 ensured that the model was well-defined over the full parameter space, including where $g \geq r$. A large grid was used with all combinations of 50 values of K, 50 values of θ, 50 values of r, and 20 values of the standard deviation of the residual errors. The number of values for each parameter included in the grid was chosen to obtain relatively smooth posterior distributions for each parameter. The posterior probability of each combination of these parameters ($n = 2.5$ million) was calculated by combining the likelihood and the priors (same priors as the brms approach above). Randomly drawing combinations of parameters from the grid weighted by their posterior probability allowed us to obtain posterior draws and posterior predictive distributions.

The posterior estimates obtained from this grid-based Bayesian approach were: $K = 0.17$ (95% credible interval: 0.16 to 0.19), $r = 0.81$ (0.77 to 0.93), and $\theta = 4.55$ (2.23 to 8.38), again indicating superlinear density dependence. There was some evidence of bimodality in the posteriors of r and θ, reinforcing the idea that the θ-logistic model is often not flexible enough to capture the true shape of density dependence, even for monotonic relationships. Irrespective of the data processing and modelling approaches used (see R

notebooks at `https://doi.org/10.5281/zenodo.20848361`), there was no qualitative change in the result; our experimental data showed superlinear density dependence.

E Density-dependence predictions for other resource dynamics

We used the parametrized Monod function (see section 1) to predict the shape of density dependence in resource supply regimes that weren't tested in our continuous-culture experiment. Median parameter estimates were used to make the predictions with posterior draws used to represent uncertainty.

To predict the shape of density dependence under chemostat resource supply (where harvest of bacteria is separated from resource dynamics), we parametrized Eq. (4) and examined how N^* changed in response to varying harvest rate (m, a "neutral parameter"), while keeping dilution rate (d) fixed.

For logistically growing resource, we started with the consumer-resource model:

$$\begin{aligned}\frac{dN}{dt} &= N\left(\frac{\mu_{\max}R}{k_S+R} - m\right)\\ \frac{dR}{dt} &= rR\left(1-\frac{R}{K}\right) - \frac{\mu_{\max}R}{k_S+R}QN\end{aligned} \tag{S21}$$

where r is the intrinsic rate of growth of the resource and K is the carrying capacity of the resource, and we could again analytically predict the shape of density dependence as:

$$N^* = \frac{k_S r[K\mu_{\max} - (k_S+K)m]}{Kq(m-\mu_{\max})^2} \tag{S22}$$

To obtain stable equilibria over a range of consumer mortality values in this model, a relatively low value of K (0.0004) and a relatively high value of r (100) were used to avoid cyclic dynamics or extinctions.

To estimate the shape of density dependence under pulsed resource dynamics we took a numerical approach, as Abrams' analytical technique is best suited to systems with fixed point equilibria. We simulated a consumer-resource system with Monod resource uptake and introduced resources as periodic pulses (with no resource loss other than through consumption). For each time step, we extracted per capita growth rate from the model and plotted this against consumer density. The magnitude and the frequency of the resource pulses has no impact on the shape of the consumer's density dependence as the resource itself has no density dependence (unlike logistic or continuous resources).

Finally, to illustrate the importance of baseline mortality rates in determining the shape of density dependence, we plotted the observed density dependence for each of the three resource supply regimes under baseline mortality rates of 25%, 50%, and 75% of the maximum growth rate.

References

[1] Jacques Monod. The growth of bacterial cultures. *Annual Review of Microbiology*, 3(1):371–394, 1949.

[2] Rui Dilao and Tiago Domingos. A general approach to the modelling of trophic chains. *Ecological Modelling*, 132(3):191–202, 2000.

[3] Richard McElreath. *Statistical rethinking: A Bayesian course with examples in R and Stan*. Chapman and Hall/CRC, 2018.

[4] Jonah Gabry, Rok Češnovar, Andrew Johnson, and Steve Bronder. *cmdstanr: R interface to 'CmdStan'*, 2025. R package version 0.9.0, https://discourse.mc-stan.org.

[5] R Core Team. *R: A language and environment for statistical computing*. R Foundation for Statistical Computing, Vienna, Austria, 2023.

[6] Bob Carpenter, Andrew Gelman, Matthew D Hoffman, Daniel Lee, Ben Goodrich, Michael Betancourt, Marcus Brubaker, Jiqiang Guo, Peter Li, and Allen Riddell. Stan: A probabilistic programming language. *Journal of Statistical Software*, 76:1–32, 2017.

[7] Peter A Abrams. Determining the functional form of density dependence: deductive approaches for consumer-resource systems having a single resource. *The American Naturalist*, 174(3):321–330, 2009.

[8] Frederick E Smith. Population dynamics in daphnia magna and a new model for population growth. *Ecology*, 44(4):651–663, 1963.

[9] Peter A Abrams. *Competition theory in ecology*. Oxford University Press, 2022.

[10] Emanuel A Fronhofer, Lynn Govaert, Mary I O'Connor, Sebastian J Schreiber, and Florian Altermatt. The shape of density dependence and the relationship between population growth, intraspecific competition and equilibrium population density. *Oikos*, page e09824, 2023.

[11] Sara A Reynolds and Chad E Brassil. When can a single-species, density-dependent model capture the dynamics of a consumer-resource system? *Journal of Theoretical Biology*, 339:70–83, 2013.

[12] Andrew D Letten. Making sense of (sublinear) density dependence. *Trends in Ecology & Evolution*, 2025.

[13] Michael E Gilpin and Francisco J Ayala. Global models of growth and competition. *Proceedings of the National Academy of Sciences*, 70(12):3590–3593, 1973.

[14] Ian A Hatton, Onofrio Mazzarisi, Ada Altieri, and Matteo Smerlak. Diversity begets stability: Sublinear growth and competitive coexistence across ecosystems. *Science*, 383(6688):eadg8488, 2024.

[15] Paul-Christian Bürkner. brms: An r package for bayesian multilevel models using stan. *Journal of Statistical Software*, 80:1–28, 2017.
